# The effect of mood state on visual search times for detecting a target in noise: An application of smartphone technology

**DOI:** 10.1371/journal.pone.0195865

**Published:** 2018-04-17

**Authors:** Toru Maekawa, Stephen J. Anderson, Matthew de Brecht, Noriko Yamagishi

**Affiliations:** 1 Center for Information and Neural Networks (CiNet), National Institute of Information and Communications Technology, Osaka, Japan; 2 School of Life and Health Sciences, Aston University, Birmingham, United Kingdom; 3 Graduate school of Frontier Biosciences, Osaka University, Osaka, Japan; 4 Department of Cognitive Neuroscience, Advanced Telecommunications Research Institute International, Kyoto, Japan; Cazenovia College, UNITED STATES

## Abstract

The study of visual perception has largely been completed without regard to the influence that an individual’s emotional status may have on their performance in visual tasks. However, there is a growing body of evidence to suggest that mood may affect not only creative abilities and interpersonal skills but also the capacity to perform low-level cognitive tasks. Here, we sought to determine whether rudimentary visual search processes are similarly affected by emotion. Specifically, we examined whether an individual’s perceived happiness level affects their ability to detect a target in noise. To do so, we employed pop-out and serial visual search paradigms, implemented using a novel smartphone application that allowed search times and self-rated levels of happiness to be recorded throughout each twenty-four-hour period for two weeks. This experience sampling protocol circumvented the need to alter mood artificially with laboratory-based induction methods. Using our smartphone application, we were able to replicate the classic visual search findings, whereby pop-out search times remained largely unaffected by the number of distractors whereas serial search times increased with increasing number of distractors. While pop-out search times were unaffected by happiness level, serial search times with the maximum numbers of distractors (n = 30) were significantly faster for high happiness levels than low happiness levels (p = 0.02). Our results demonstrate the utility of smartphone applications in assessing ecologically valid measures of human visual performance. We discuss the significance of our findings for the assessment of basic visual functions using search time measures, and for our ability to search effectively for targets in real world settings.

## Introduction

The emotional status of individuals affects not only their creative abilities [1–3] and capacity for social interaction [4, 5], but also their ability to perform various sensory-motor [6] and cognitive tasks [7]. The broad range of human abilities affected by mood is consistent with the belief that the evolutionary adaptive value of positive emotions extends from an enhancement of high-level personal resources (e.g. intellectual capacity) to low-level physiological support for survival in life-or-death situations [8].

Emotions are short-lived experiences which produce coordinated changes in an individual’s behavioral and physiological responses [9–11]. Using film clips to momentarily alter mood state, Fredrickson and Branigan [11] assessed the effect of mood on human vision, demonstrating that positive emotions yield a bias for perceiving global over local configural aspects of a visual target. Similar findings were reported by Gasper and Clore [12], who concluded that positive mood states foster global visual processing. To explain these results, Fredrickson and Branigan hypothesized that positive emotions broaden attentional, cognitive and action processes while negative emotions narrow these same processes.

Although various electrophysiological [13, 14], eye movement [15] and psychophysical studies [16–18] on human vision provide evidence in general support of a broadening of attentional allocation with positive emotions, other studies that have employed similar methodological paradigms have failed to show such effects (see [19, 20]).

Tentative explanations that have been advanced to explain the discrepant results reported above include the sample size of individual experiments, motivational salience of positive information, and the relevance of peripheral information in global-local visual choice tasks [19, 20]. Another possible explanation for the discrepant results between studies on mood state and visual processing lies in the wide variety of emotion induction methods employed, which include film clips, music, vocal expressions, visual images, food, mental imagery, and the viewing of positive or negative valenced words. That is not to say that such methods are ineffective, but simply that different mood induction methods may yield different results. Indeed, even a single mood-inducing method, such as music, can produce a range of emotional responses in different individuals [21]. Finally, it is axiomatic that the ability to maintain a particular mood state will be dependent on the induction method employed, which may be especially significant if it is the case that emotions are short-lived experiences [11, 22].

In brief, while it is clear that an individual’s creativity and capacity for social interaction are influenced by their emotional state, it remains an open question as to what extent mood alone can influence low-level visual processes. Our principal aim in this study was to determine whether rudimentary visual search processes are affected by the emotional status of an individual. Specifically, we examined whether the level of happiness perceived by an individual could affect their ability to detect a visual target in noise.

To overcome the inherent problems associated with the use of experimental mood induction procedures [22], we developed a novel Smartphone application that allowed us to collect visual search data (search times) and self-rated levels of happiness throughout each twenty-four-hour period for two weeks, affording us the opportunity to assess everyday emotional changes within an individual’s normal home/work environment. In developing our Smartphone application, we were guided by the belief that the most reliable method for assessing real-world emotion is experience sampling [23], whereby individuals are required to report their thoughts, feelings and actions as they go about their everyday activities. Indeed, we note that Smartphone technology has recently been successfully exploited by cognitive scientists as a medium for assessing the mental health of students [24], working memory and decision making [25–27], momentary subjective well-being [28], age-related changes in decision making [29] and proactive and reactive control with aging [30].

Validation of our particular Smartphone application for use in visual search experiments was achieved by our ability to replicate the results of Treisman’s [31] classic search paradigms, in which pop-out search times remain largely unaffected by the number of distractors while serial search times increase with increasing number of distractors. We go on to show that serial search times may be significantly affected by an individual’s emotional status.

## Methods

### Participants and ethics statement

An a priori power analysis in G*Power [32, 33] for a three-way repeated measures ANOVA with a power = 0.8, a medium effect size = 0.3 and a sphericity correlation value = 0.7 indicated a minimum sample size of 21 participants. In total, 33 individuals gave informed written consent and were recruited for the study (25 male, 8 female, aged 20–35 years). All participants had normal or corrected-to-normal visual acuity and normal color vision. To promote compliance and data quality, all were offered a modest monetary reward on completion of the study. Approval for the study was obtained from the Ethics Committee for Human and Animal Research (National Institute of Information and Communications Technology), and all procedures involving human subjects were in accordance with the tenets of the Declaration of Helsinki.

### Smartphone application and experimental design

**Fig 1:**
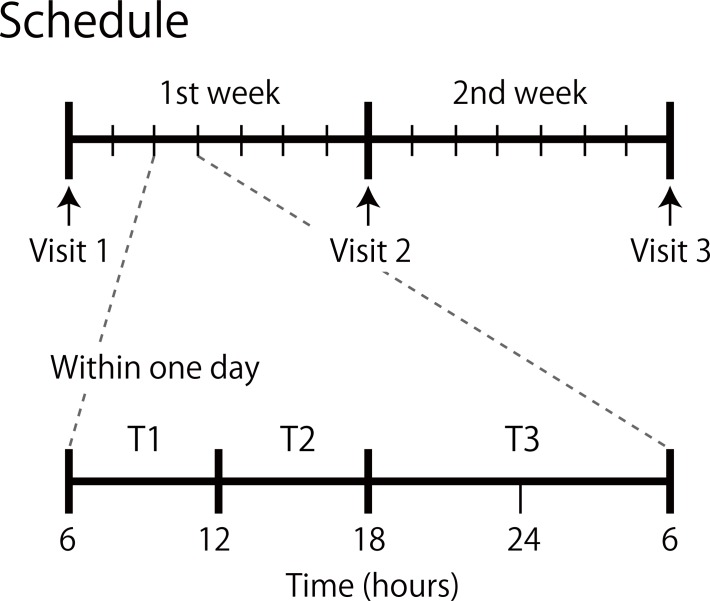
Experimental schedule. Each participant was asked to complete both pop-out and serial visual search tasks three times daily [at any time between the set periods 6am to 12 noon (T1), 12 noon to 6pm (T2), and 6pm to 6am the following day (T3)] for fourteen days. An expanded time sequence is shown for the third day of the schedule.

Participants were asked to complete both pop-out and serial visual search tasks three times daily (at any time between the set periods 6am to 12 noon, 12 noon to 6pm, and 6pm to 6am the following day) for fourteen days (see Fig 1), with all measures completed within their normal home/work environment. Prior to completing the visual search tasks, participants were required to complete a motor response touch task and self-rate their level of happiness. This in-situ probing of happiness level was augmented with other self-rated measures of the participants’ current circumstances (sleepiness, level of stress, extent of smartphone usage and face-to-face communication). Procedural details of these various tasks are given below.

To implement this experimental design an application on iOS (Apple Inc.), termed *VisualSearch*, was built by the first author (TM) and installed on each participant’s iPhone during their initial visit to the laboratory. Each iPhone model used (models 4–7 inclusive) had a touch sampling rate of 60 Hz, and each displayed and ran the developed app in the same manner. At that first visit, each participant was instructed on how to use the Smartphone application, and their visual acuity and colour vision were assessed. At the second visit, approximately one week later, data from each subject’s Smartphone was collected and assessed to ensure the application was functioning correctly. At the final visit, after two weeks, all data was collected and the search-task application was uninstalled. Note that validation of *VisualSearch* was done prior to and independently of the reported experiments using five participants who were not part of the main study. The source code for *VisualSearch* is freely available upon request.

**Fig 2:**
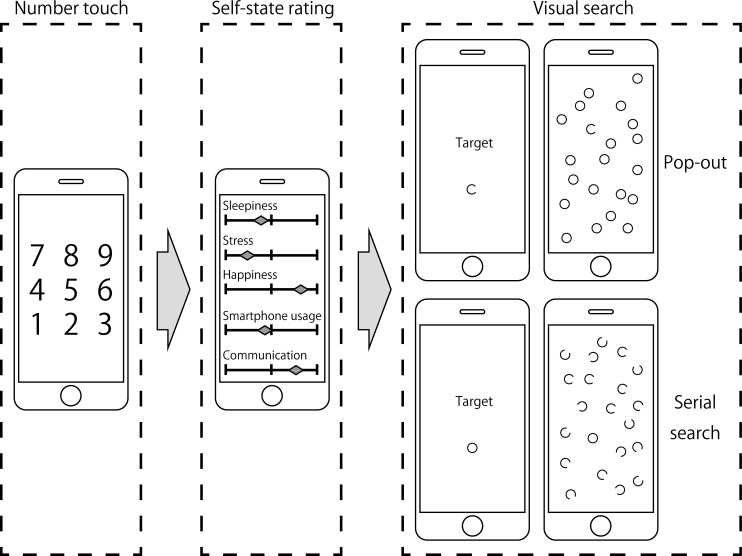
Schematic view of the smartphone displays on each task. The left panel shows the number touch task. The middle panel shows the self-state rating task. The right panels show, for the number of distractors set equal to 20, the serial and pop-out visual search tasks. See text for further explanation.

Fig 2 provides a representation of the Smartphone display images associated with the motor response number touch task, the self-state rating task and the pop-out and serial visual search tasks. Completing all of the tasks in a single recording session took approximately five minutes. All participants were instructed to hold the Smartphone in their non-dominant hand, and to respond by tapping the face of the phone with the index finger of their dominant hand. The maximum number of sessions that could be completed was 42 (three sessions per day for 14 days). Although there was no Smartphone alert to prompt a recording session, missed sessions were rare: at the completion of the study, an average of 40.3 ±0.55 (± 1 s.e.) sessions were completed (range 30–42 sessions, mode 42 sessions).

### Procedures

#### Motor response number touch task

The purpose of this task was to check whether mood state affects motor response speed. Following the tapping of a start button, the numbers one to nine were displayed as blue characters on a white background (see Fig 2). The digits appeared in the same order during each session in order to avoid any confound between motor response speed and search time differences. Participants were instructed to tap the numbers in sequence from one to nine as quickly as possible, with response time recorded as the time interval between tapping the first and the last number. When correctly tapped, the colour of the text character changed from blue to red.

#### Self-state rating task

Our principal measure was the participants’ level of happiness, which was rated using a non-integer visual analogue sliding scale that ranged from zero (‘Not at all happy’) to 10 (‘Very happy’) [34, 35]. To gain some understanding of the participants’ current circumstances, and to help disguise happiness level as our principal measure, each participant was also asked to rate their present levels of sleepiness (‘not at all’ to ‘very’), stress (‘none’ to ‘high’) and social interactions (‘none’ to ‘high’). With regard to the latter, participants were instructed to consider the extent of their social interactions through Smartphone usage and/or face-to-face communications between the previous and current recording sessions. Depression of an on-screen start button caused the rating topics and slider axes to be displayed (see Fig 2). To help minimize any survey bias, scale pointers were not displayed at the beginning of each measure but instead were made to appear once a participant had tapped a particular scale. Each pointer could then be dragged to an appropriate physical location on the scale to reflect the participant’s self-rated measure: participants were aware that the hard-left of the scale signified ‘not at all’ or ‘none’ and that the hard-right of the scale signified ‘very’ or ‘high’. Note that the actual numerical value (real number between zero and 10) was not displayed on-screen. After the self-rated measure for each scale had been set, participants tapped an on-screen button to proceed to the visual search task.

#### Visual search tasks

Using stimulus arrays similar to those employed by Treisman and Souther [36], participants were required to complete both pop-out and serial visual search tasks. In the pop-out task, the single target was a circle with a gap in it (‘open circle’) and the distractors were complete circles (‘closed circles’). In the serial search task, the single target was a complete circle and the distractors were circles with gaps. The radius of the circles, both open and closed, was 13 pixels. The size of the gap in the open circles was approximately one tenth the circumference of the closed circles. The location of the gap in the open circles was random. Fig 2 gives a schematic representation of the Smartphone display for each search type.

Participants completed two search types (serial and pop-out) for each distractor condition (10, 20 and 30 distractors) to give a total of six trials per session. The order of conditions within each session was random, with the location of the target and distractors randomized between trials.

Each trial began with a presentation of what the target would be. On depression of an on-screen start button, the search display was presented and participants were required to locate and tap the target with their index finger as quickly as possible. After tapping the target the participants’ search time, defined as the time between tapping the start button and the target, was shown. The target for the next trial was then displayed. Note that incorrect response trials numbered less than 1.0% of the total responses, and were excluded from further analyses.

## Results

For each participant, motor response speeds and visual search times that exceeded three standard deviations from the mean in each experimental condition numbered less than 1.5% of trials and were excluded from the analyses reported below.

### Serial and pop-out search times recorded with smartphone technology

**Fig 3:**
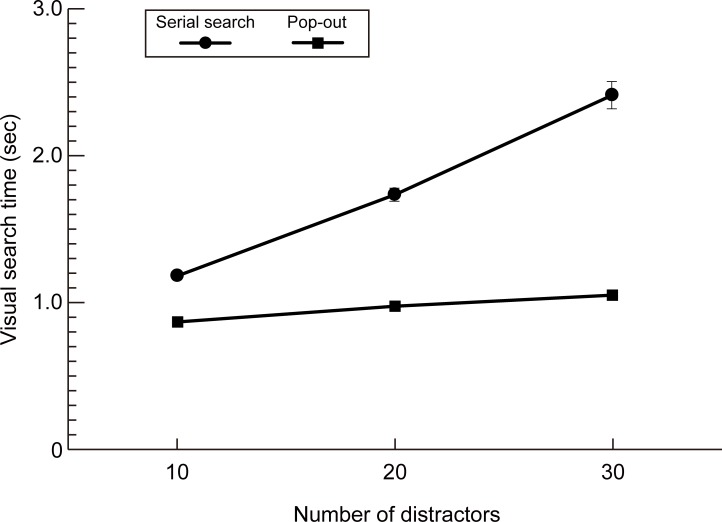
Serial and pop-out search times. Line diagram showing the mean (n = 33) visual search times for three distractor conditions (10, 20 or 30 distractors) for both serial (closed circles) and pop-out (closed squares) search tasks. Error bars show ± one standard error of the mean. Note that for some conditions, the error bars are not visible because one standard error was less than or equal to the symbol size.

The mean (n = 33) visual search times for three distractor conditions (10, 20 or 30 distractors) are shown in Fig 3 for both serial (closed circles) and pop-out (closed squares) search tasks. Note that as the number of distractors increased, serial search times increased while pop-out search times remained largely unchanged, results that are in agreement with those obtained under laboratory conditions by Treisman and Souther [36]. A least-squares fit of a straight line to each data set yielded a slope of 57.3 msec per distractor for the serial search task and 8.8 msec per distractor for the pop-out task. A two-way repeated measures ANOVA revealed significant main effects for visual search type (F(1, 32) = 356, p < 0.0001) and the number of distractors (F(2, 64) = 248, p < 0.0001), and a significant interaction between search type and the number of distractors (F(2, 64) = 133, p < 0.0001). Note that error rates did not differ between visual search types (F(1, 32) = 1.00, p = 0.33), the number of distractors (F(2, 64) = 2.47, p = 0.09) or the interaction between search types and the number of distractors (F(2, 64) = 49, p = 0.61).

To assess whether session time affected the results, the search time data were divided into three groups based on session times T1, T2 and T3 (see Fig 1). A three-way repeated measures ANOVA revealed no interaction between the number of distractors, search type and session time (F(2, 128) = 1.19, p = 0.32; see S1 File).

### The effect of happiness levels on visual search times

To assess the effect of mood on visual search times, happiness ratings were first transformed into Z scores for each participant (n = 33) in order to account for their average mood level and intra-individual variability. The Z scores were then divided into three subgroups: scores < -0.5 were defined as low happiness levels; scores between -0.5 and 0.5 were defined as moderate; and scores > 0.5 were defined as high [37]. Table 1 shows the mean (n = 33) visual search times (seconds) for each subgroup, for both search types and for each distractor condition.

**Table 1:** Pop-out and serial visual search times.

Search type	Distractor condition	Happiness level					
		Low		Moderate		High	
		z-score: < -0.5		z-score: -0.5 to 0.5		z-score: > 0.5	
		Mean (s)	s.e.	Mean (s)	s.e.	Mean (s)	s.e.
**Pop-out**	10	0.854	0.021	0.849	0.013	0.834	0.016
	20	0.974	0.017	0.947	0.019	0.934	0.017
	30	1.020	0.023	1.020	0.021	1.038	0.024
**Serial**	10	1.183	0.038	1.154	0.029	1.106	0.034
	20	1.679	0.053	1.638	0.048	1.740	0.065
	30	2.484	0.135	2.326	0.095	2.200	0.081

Visual search times (seconds, s) and standard errors (s.e.) for low (z < -0.5), moderate (z = -0.5 to 0.5) and high (z > 0.5) happiness levels, for both pop-out and serial search types and for each distractor condition (10, 20 or 30 distractors).

**Fig 4:**
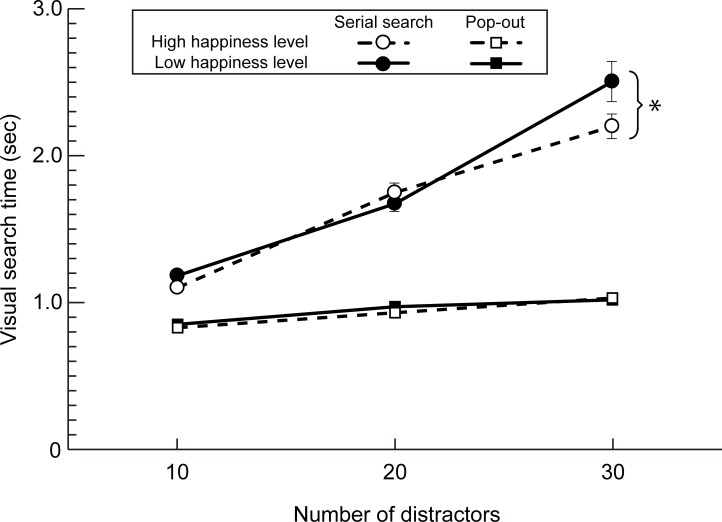
Effect of happiness level on search times. Line diagram showing the mean (n = 33) serial (circles) and pop-out (squares) search times for each distractor condition for both low (solid symbols) and high happiness (open symbols) levels. Error bars show ± one standard error of the mean. Note that for some conditions, one standard error was less than or equal to the symbol size.

Fig 4 shows the mean serial (circles) and pop-out (squares) search times for each distractor condition for both the low (solid symbols) and high happiness (open symbols) levels, as defined above. A three-way repeated measures ANOVA revealed significant main effects for search type (F(1, 32) = 296, p < 0.0001) and distractor condition (F(2, 64) = 208, p < 0.0001), and a significant interaction between search type, distractor condition and happiness level (F(2, 64) = 6.59, p = 0.003, η^2^ = 0.17). Post-hoc analyses with Shaffer’s method [38] indicated that, for the condition in which 30 distractors were used, the serial visual search time for the high happiness level was significantly faster than that for the low happiness level (F(1, 32) = 6.12, p = 0.02, η^2^ = 0.16). Note that this pattern of results held for statistical analyses performed with log-transformed visual search times, completed because of the differing magnitude of standard errors between distractor conditions (see S2 File).

Incorporating all the data (i.e. low, moderate and high happiness levels), a three-way repeated measures ANOVA again revealed a significant interaction between search type, distractor condition and happiness level (F(4, 128) = 3.53, p = 0.01, η^2^ = 0.10). Post-hoc analyses with Shaffer’s method revealed, for the 30 distractor condition alone, that there was a significant effect of happiness on serial visual search times (F(2, 64) = 3.49, p = 0.04, η^2^ = 0.10). While there was a significant difference between high and low happiness levels (t(32) = 2.31, p = 0.03, Cohen’s d = 0.44), there was no significant difference between either the high and moderate happiness levels (t(32) = 1.45, p = 0.15, Cohen’s d = 0.25) or between the moderate and low happiness levels (t(32) = 1.40, p = 0.17, Cohen’s d = 0.24).

### Multiple linear regression analysis of self-state ratings

The serial search data obtained with 30 distractors was analyzed further using hierarchical linear modeling [39] to determine the association between visual search time and participants’ self-rated measures of sleepiness, stress, smartphone usage, direct communication and happiness. Our data represent a two-level structure, with sessions (Level 1; n = 1331) nested within participants (Level 2; n = 33). Visual search time was assessed as the dependent variable, and the independent (‘predictor’) variables were the five self-rated measures. The analyses were implemented using the software package IBM SPSS Statistics version 24.

**Fig 5:**
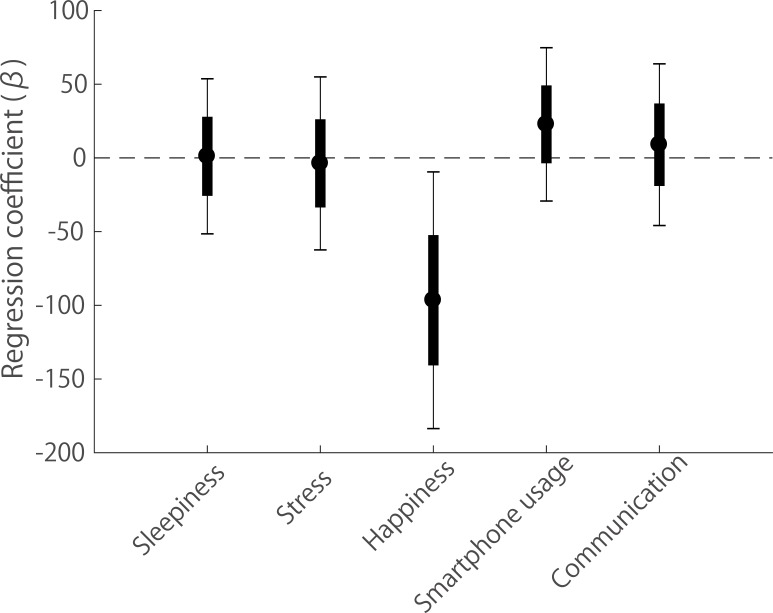
Multiple linear regression analysis. Regression coefficients (β, solid circles), standard errors (± 1 s.e., solid rectangles) and 95% Confidence Intervals (CI, thin vertical lines) for the five predictors (sleepiness, stress, happiness, Smartphone usage and communication) of serial visual search times.

Fig 5 shows the regression coefficient (β, solid circle) associated with each independent variable. The coefficient values indicate the degree of unit change in serial visual search time (msec) for each unit increase in a particular variable, given that all other variables are held constant. The solid rectangle and thin vertical lines extending from each regression coefficient represent ± one standard error and the 95% confidence intervals, respectively. Note that the regression coefficient for ‘happiness’ is significantly below zero (i.e. null effect) (happiness β = -96.56, t = -2.18, p = 0.03), indicating that participants’ achieved faster serial visual search times when they reported their happiness level to be high. Assuming the other variables (sleepiness, stress, smartphone usage and communication) are constant, we would predict a 96.56 msec decrease in serial search time for a one unit increase in a participant’s happiness level (range: 0–10). Note that none of the remaining variables had a significant effect on visual search time (sleepiness β = 1.09, t = 0.041, p = 0.97; stress β = -3.72, t = -0.12, p = 0.90; smartphone usage β = 22.77, t = 0.86, p = 0.39; communication β = -8.97, t = 0.32, p = 0.75).

### Effects of session time on self-state ratings

**Fig 6:**
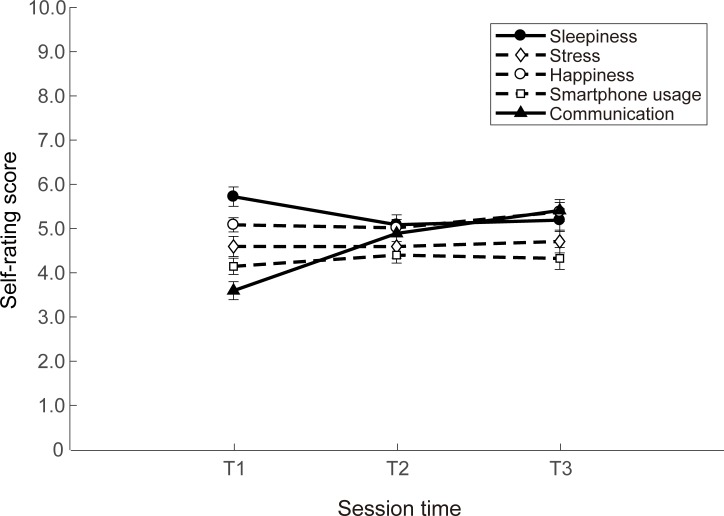
Effect of session time on self-state ratings. The mean self-state rating scores (across all distractor conditions) for sleepiness (solid circles), stress (open diamonds), happiness (open circles), smartphone usage (open squares) and communication (closed triangles), plotted for session times T1 (6am to 12 noon), T2 (12 noon to 6 pm) and T3 (6 pm to 6 am the following day). The vertical error bars show ± one standard error of the mean.

Fig 6 shows, for session times T1, T2 and T3 (from Fig 1), the average self-state rating scores (across all distractor conditions) for sleepiness, stress, happiness, smartphone usage and communication. Note that the score for sleepiness (solid circles) was highest during the morning session (T1, 6 am to 12 noon), while the communication score (solid triangles) was lowest during this session. In assessing whether session time affected participants’ self-state ratings, a one-way repeated measures ANOVA revealed a significant interaction between session time and both sleepiness (F(2, 64) = 5.87, p = 0.02, η^2^ = 0.16) and communication (F(2, 64) = 23.92, p < 0.001, η^2^ = 0.43). There was no significant interaction between session time and the self-state ratings of stress (F(2, 64) = 0.62, p = 0.54), happiness (F(2, 64) = 3.20, p = 0.15) or smartphone usage (F(2, 64) = 1.00, p = 0.74). All reported p values were corrected for multiple comparisons by Holm’s method [40], which was used to control the family-wise error rate.

### Mood state and motor response speed

In a control study, we assessed whether mood state (happiness level) affects motor response speed using a speeded number touch task (see Methods). As in the analysis of the visual search data, happiness ratings were transformed into z scores for each participant (n = 33) and then divided into three subgroups: scores < -0.5 were defined as low happiness levels; scores between -0.5 and 0.5 were defined as moderate; and scores > 0.5 were defined as high. The motor response speed (± se) was 3.06 s ± 0.18 s for the high happiness level and 3.10 s ± 0.20 s for the low happiness level, a difference that was not significant (F(1, 32) = 0.37, p = 0.55).

## Discussion

In this paper we sought to determine whether mood can affect basic measures of visual performance, in particular visual search times. To do so we used the technique of experience sampling [23] to collect visual search data and self-rated levels of happiness over a two week period, implemented with a novel Smartphone app. Our aim with this approach was to avoid the drawbacks associated with laboratory methods for inducing temporary mood states [22] in favour of assessing the effects of mood within an individual’s normal environment. General support for this approach, including the Smartphone app, was provided by our initial experiment in which we were able to replicate the classic serial/pop-out visual search dichotomy first reported by Treisman [31] (see Fig 3). We went on to show that, while pop-out search times are unaffected by mood, serial search times are significantly faster for high happiness levels than low happiness levels (Figs 4 and 5, and S2 File). In contrast, we did not find any significant relationship between search times and self-rated measures of perceived sleepiness, stress level or the extent of social interactions (see Fig 5).

In comparison with a conventional laboratory setting for psychophysical experiments, experience sampling using Smartphone technology necessarily requires data collection within uncontrolled testing environments. Nonetheless, consistent with the reported successful use of Smartphones in assessing cognitive abilities (e.g. [24, 27, 29]), physiological functions [41, 42] and auditory processes [43], our results demonstrate the potential use of Smartphone applications for psychophysical measures of human visual performance. In particular, we suggest that Smartphones are a valid, convenient and cost-effective means of assessing the effect of everyday mood changes on the ability of individuals to perform visual searches (see also [44]).

Visual searches are essential for all animal species with foveated systems. In humans, they are an indispensable part of life and include everything from mundane searches for products on supermarket shelves through to frenzied searches for a lost child at a fairground. Indeed, measures of search times for detecting targets in noise–generally completed within conventional laboratory settings–have long been used as a means of investigating various aspects of human perception and cognition, including visuo-motor actions, spatio-temporal integration of information and attention. Factors known to influence visual search response times include retinal inhomogeneity, crowding, memory, statistical properties of target/distractors, contextual cues and scene context (reviewed in Eckstein [45]). Our results provide evidence that the emotional status of individuals must also be added to this list, given that we show serial search times to be significantly faster during happy mood states.

While we were principally concerned with the effects of mood state, we also determined the relationship between search time and participants’ self-rated measures of their current circumstances (Fig 5). Of all the factors considered, one might expect that search times would be positively correlated with perceived levels of sleepiness, as cognitive performance is known to decline with fatigue [46, 47]. However, we did not find any significant relationship between search time and sleepiness. This is consistent with the findings of Takahashi et al. [48], who reported that reaction time measures recorded within an individual’s normal work environment were uncorrelated with their perceived level of alertness.

The mechanism by which mood affects visual perception remains unclear, though the results of most studies are consistent with the notion that positive mood states broaden attentional processes and in consequence yield a bias for perceiving global over local configural aspects of a visual target (e.g. [10–12, 17, 49]). Our results may be considered within this general framework if one supposes that high happiness levels cause a broadening or strengthening of the attentional field, thereby enhancing performance for detecting the target stimulus across a wider area of the retina.

Whether this is the case or not, our data showing that visual searches are affected by emotion is important because it highlights the deleterious effects mood can have on our ability to search effectively for targets in real world settings. Perhaps the field of radiology is a prime example of this, populated as it is by a group of people who might aptly be called ‘professional searchers’. The ever-increasing case load and rapid reporting requirements of radiologists are known to affect their performance [50–52], with an estimated day-to-day diagnostic error rate of 3–5% [53], the bulk of which are perceptual errors [50, 54]. While perceptual errors are likely to reflect a range of factors, including case complexity, non-standard imaging protocols and observer fatigue [52], our results suggest that the level of such errors may also be dependent on the radiologist’s mood. As far as we are aware, the latter is unrecognized as a contributing factor to diagnostic errors in radiology.

## Supporting information

S1 File Effect of session time on visual search time. (PDF)

S2 File Effect of happiness level on log-transformed visual search times. (PDF)
