# Supplementary material for: The effect of mood state on visual search times for detecting a target in noise: An application of smartphone technology
Source: PLoS One. 2018 Apr 17;13(4):e0195865. doi: 10.1371/journal.pone.0195865 (PMC5903627; doi:10.1371/journal.pone.0195865)

S1 File. **Effect of session time on visual search time.** Line diagram showing the mean (n = 33) visual search times (sec), for both serial (circles) and pop-out (squares) search tasks. Results are shown for each distractor condition, for session times T1 (6 am to 12 noon, black symbols), T2 (12 noon to 6 pm, gray symbols) and T3 (6 pm to 6 am the following day, open symbols). Error bars show ± one standard error of the mean. Note that for some conditions, the error bars are not visible because one standard error was less than or equal to the symbol size.

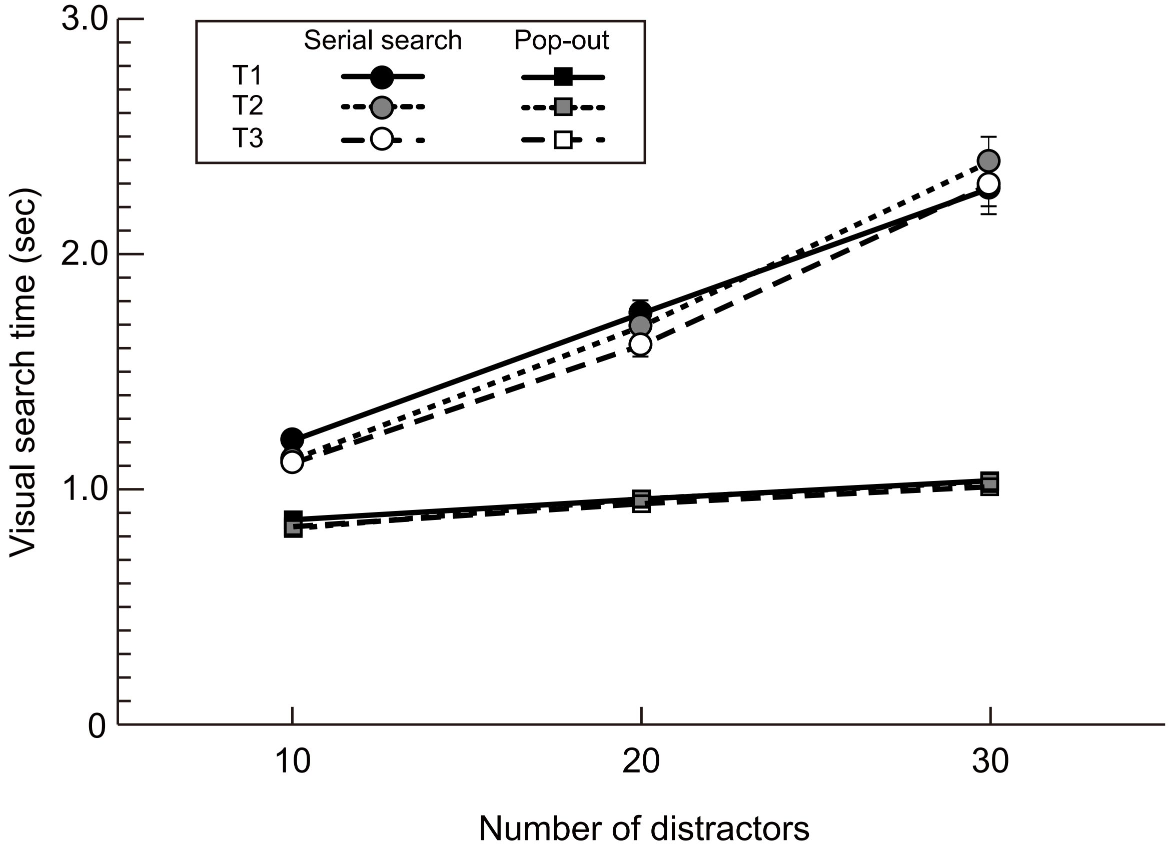

Supplement: S1 File — (PDF) [file pone.0195865.s001.pdf]
