# Supplementary material for: The effect of mood state on visual search times for detecting a target in noise: An application of smartphone technology
Source: PLoS One. 2018 Apr 17;13(4):e0195865. doi: 10.1371/journal.pone.0195865 (PMC5903627; doi:10.1371/journal.pone.0195865)

S2 File. **Effect of happiness level on log-transformed visual search times.** Line diagram showing the mean (n = 33) serial (circles) and pop-out (squares) log-transformed visual search times for each distractor condition for both low (solid symbols) and high happiness (open symbols) levels. Error bars show ± one standard error of the mean. Note that for some conditions, the error bars are not visible because one standard error was less than or equal to the symbol size. A three-way repeated measures ANOVA revealed a significant interaction between search type, distractor condition and happiness level ($F(2, 64) = 5.34$, $p = 0.007$, $\eta^2 = 0.14$). Post-hoc analyses with Shaffer's method for the 30 distractor condition indicated that the serial visual search time for the high happiness level was significantly faster than that for the low happiness level ($F(1, 32) = 6.64$, $p = 0.01$, $\eta^2 = 0.17$).

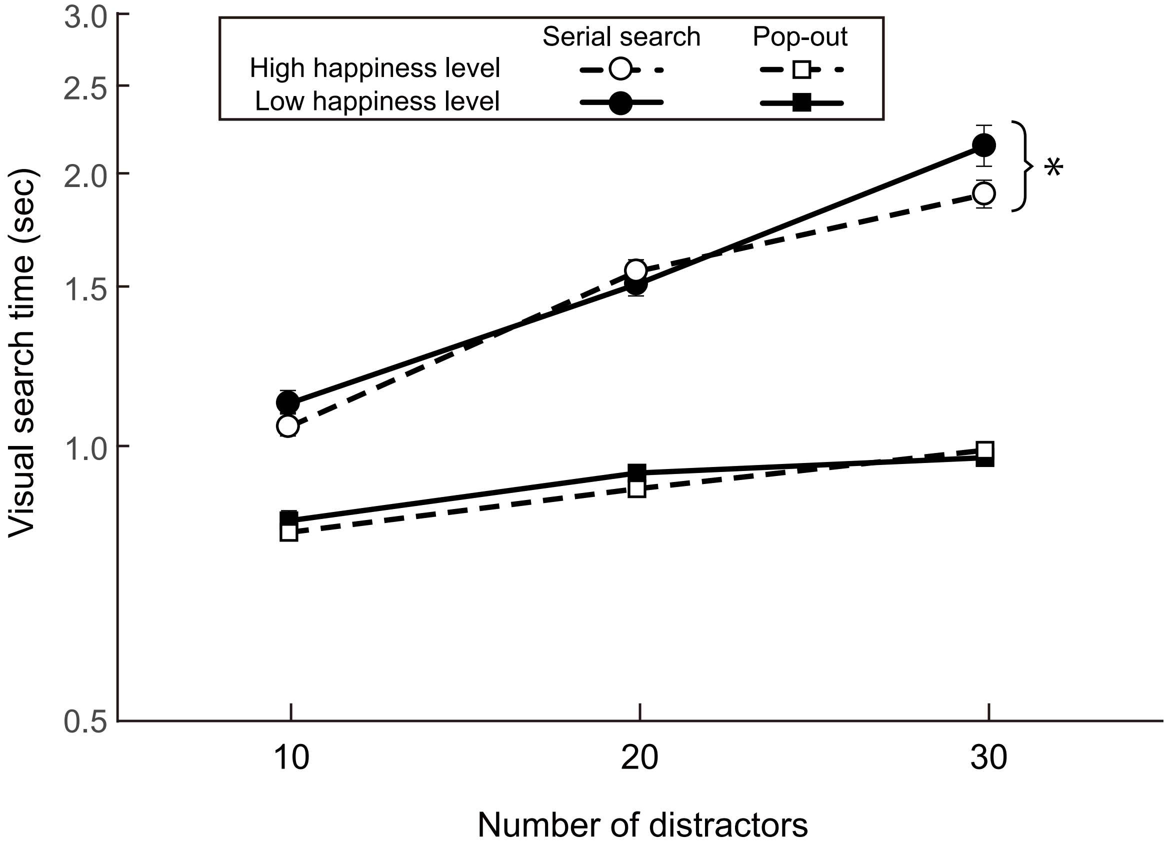

Supplement: S2 File — (PDF) [file pone.0195865.s002.pdf]
